# Construction of Boronophenylalanine-Loaded Biodegradable Periodic Mesoporous Organosilica Nanoparticles for BNCT Cancer Therapy

**DOI:** 10.3390/ijms22052251

**Published:** 2021-02-24

**Authors:** Fuyuhiko Tamanoi, Shanmugavel Chinnathambi, Mathilde Laird, Aoi Komatsu, Albane Birault, Takushi Takata, Tan Le-Hoang Doan, Ngoc Xuan Dat Mai, Arthur Raitano, Kendall Morrison, Minoru Suzuki, Kotaro Matsumoto

**Affiliations:** 1 Institute for Integrated Cell-Material Sciences, Institute for Advanced Study, Kyoto University, Kyoto 606-8501, Japan; chinnathambi.shanmugavel.8s@kyoto-u.ac.jp (S.C.); laird.mathilde.3e@kyoto-u.ac.jp (M.L.); komatsu.aoi.6z@kyoto-u.ac.jp (A.K.); albane06@gmail.com (A.B.); matsumoto.kotaro.5r@kyoto-u.ac.jp (K.M.); 2 Institute for Integrated Radiation and Nuclear Science, Kyoto University, Kumatori 590-0494, Japan; takata.takushi.6x@kyoto-u.ac.jp (T.T.); suzuki.minoru.3x@kyoto-u.ac.jp (M.S.); 3 Center for Innovative Materials and Architectures (INOMAR), Vietnam National University, Ho Chi Minh City 721337, Vietnam; dlhtan@inomar.edu.vn (T.L.-H.D.); mnxdat@inomar.edu.vn (N.X.D.M.); 4 TAE Lifesciences, Drug Development Division, Santa Monica, CA 90404, USA; araitano@taelifesciences.com (A.R.); kmorrison@taelifesciences.com (K.M.)

**Keywords:** biodegradable periodic mesoporous organosilica, boronophenylalanine, boron neutron capture therapy, cancer cells, chicken egg tumor model

## Abstract

Biodegradable periodic mesoporous organosilica (BPMO) has recently emerged as a promising type of mesoporous silica-based nanoparticle for biomedical applications. Like mesoporous silica nanoparticles (MSN), BPMO possesses a large surface area where various compounds can be attached. In this work, we attached boronophenylalanine (^10^BPA) to the surface and explored the potential of this nanomaterial for delivering boron-10 for use in boron neutron capture therapy (BNCT). This cancer therapy is based on the principle that the exposure of boron-10 to thermal neutron results in the release of α-particles that kill cancer cells. To attach ^10^BPA, the surface of BPMO was modified with diol groups which facilitated the efficient binding of ^10^BPA, yielding ^10^BPA-loaded BPMO (^10^BPA-BPMO). Surface modification with phosphonate was also carried out to increase the dispersibility of the nanoparticles. To investigate this nanomaterial’s potential for BNCT, we first used human cancer cells and found that ^10^BPA-BPMO nanoparticles were efficiently taken up into the cancer cells and were localized in perinuclear regions. We then used a chicken egg tumor model, a versatile and convenient tumor model used to characterize nanomaterials. After observing significant tumor accumulation, ^10^BPA-BPMO injected chicken eggs were evaluated by irradiating with neutron beams. Dramatic inhibition of the tumor growth was observed. These results suggest the potential of ^10^BPA-BPMO as a novel boron agent for BNCT.

## 1. Introduction

Biodegradable periodic mesoporous organosilica (BPMO) nanoparticles are a special type of mesoporous silica nanoparticles (MSN) that contain biodegradable chemical bonds such as di- and tetrasulfide bonds within the framework of the nanoparticle [1,2,3,4,5,6]. These nanoparticles have enhanced degradation under reducing conditions such as that encountered inside cells. Like MSN, BPMO nanoparticles have thousands of pores and thus possess a large surface area to which a variety of compounds can be attached [1,4]. They are also amenable to various chemical modifications so that surface properties can be altered [1,4]. Previously, we, as well as others, have used BPMO as a delivery vehicle for anticancer drugs such as doxorubicin and daunorubicin [7,8].

In this study, we set out to explore the use of BPMO to deliver boron-10 that could be used in boron neutron capture therapy (BNCT), a powerful cancer therapy that relies on using neutron exposure of boron-10 [9,10,11,12,13]. Since boron-10 can be split into a helium nucleus and recoiling lithium ion upon neutron irradiation, this reaction can result in killing cancer cells due to the high energy of the α−particles (helium nuclei) and their ability to cause irreparable double-stranded DNA breaks. BNCT was first proposed by Locher in 1936 [13], and a variety of clinical studies against brain cancer, head and neck tumors and others were carried out using neutrons generated at nuclear reactors. Use of boron-10 compounds such as BPA (boronophenylalanine) and BSH (mercaptoundecahydrododecaborate) advanced BNCT therapy yielding promising results [10,12]. BPA was particularly effective, as this amino acid analogue exhibits selective tumor accumulation due to increased uptake of amino acids by cancer cells. Accelerator-based neutron generators have recently been developed, bringing BNCT into hospital settings [12]. Thus, BNCT is expected to become a widely used cancer therapy in the future. 

One of the key issues regarding BNCT is to achieve tumor accumulation of boron-10. While a certain level of tumor accumulation can be achieved with BPA, further improvement is desirable. Recently, new types of boron reagents, including nanoparticle formulations of the boron-10 drug, have been reported [14,15,16,17]. While BPA is not toxic, a relatively large amount of the drug needs to be administered, and continuous infusion over 2–3 h is used to administer BPA. Nanoparticle-based drugs, with the potential to deliver more boron, could be administered as a single IV bolus injection. This is highly desirable in a hospital setting. 

To load BPA onto BPMO, we developed a method that modifies nanoparticle surface with diols, which are then used to capture boron. This method was initially used for preparing MSN filters that could remove boron from contaminated water [18]. We show in this paper that BPA-loaded BPMO nanoparticles can be efficiently synthesized using this method. To examine how the prepared BPA-BPMO behaves in biological systems, we used cancer cells, which demonstrated efficient uptake into cancer cells and perinuclear localization. We also used the chicken egg tumor model, a convenient and versatile animal model that has been used for the characterization of nanoparticles [19,20,21,22,23,24,25,26]. This model uses fertilized eggs, and the tumor is produced inside the egg on the chorioallantoic membrane (CAM). We observed significant tumor accumulation of the BPA-loaded BPMO nanoparticles. This encouraged us to irradiate the chicken eggs with the neutron beam at the Kyoto University Research Reactor in Kumatori, Japan. The results demonstrate significant tumor growth inhibition. 

## 2. Results

### 2.1. Synthesis and Characterization of BPMO Nanoparticles

Biodegradable periodic mesoporous organosilica (BPMO) nanoparticles represent attractive materials for biomedical applications [1]. They are designed to undergo degradation upon encountering conditions such as redox and low pH conditions. For this study, we chose to use BPMO that contain tetrasulfide bonds in the framework. We have previously shown that these nanoparticles are degradable by reducing conditions such as the presence of glutathione (GSH) [8]. 

**Figure 1:**
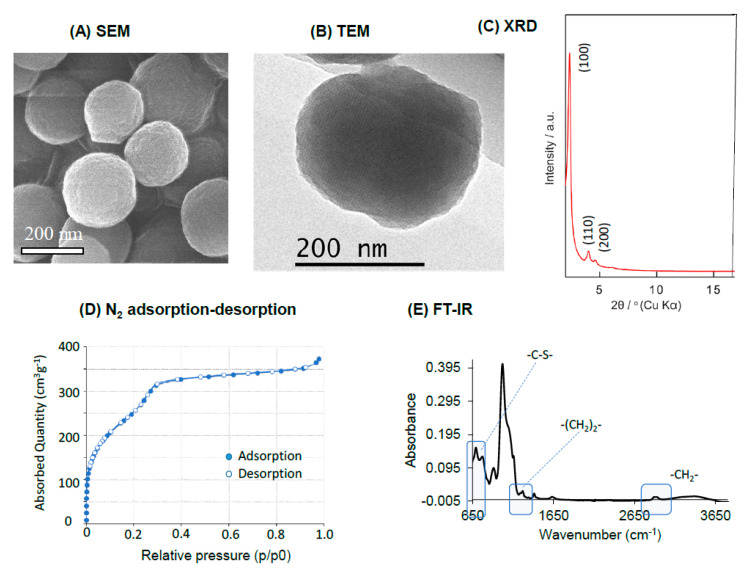
Characterization of biodegradable periodic mesoporous (BPMO) particles. (**A**,**B**) SEM and TEM pictures; (**C**) XRD analysis. (**D**) Nitrogen adsorption–desorption analysis; (**E**) FT-IR analysis.

The synthesis of BPMO was carried out using bis[3-(triethoxysilyl) propyl] tetrasulfide and 1,2-bis(triethoxysilyl)ethane as precursors of BPMO synthesis, which results in the incorporation of tetrasulfide bonds into the framework of the nanoparticle. Rhodamine-B labeling was carried out for the detection of the nanoparticles by fluorescence. In addition, phosphonate modification was carried out to increase the dispersibility of the nanoparticle [8]. Scanning Electron Microscopy (SEM) and Transmission Electron Microscopy (TEM) characterization of BPMO shown in Figure 1A,B show uniform particles with a diameter of approximately 200 nm. Dynamic light scattering analysis (DLS) shows that the diameter is 170 nm (Figure 1A: Control). The mesopore structures are seen in the TEM analysis. XRD analysis (Figure 1C) shows a typical 2D-hexagonal periodic feature of BPMO. The nitrogen adsorption–desorption analysis shown in Figure 1D reveals a type IV isotherm with parallel adsorption and desorption branches. Based on this analysis, we calculate that the surface area is 985.96 m^2^·g^−1^, pore volume is 0.577 cm^3^·g^−1^, and the pore diameter is 2.34 nm. Fourier transform infrared (FT-IR) analysis is shown in Figure 1E. In addition to a typical Si-O-Si at around 1000 cm^−1^, we observed peaks that represent -C-S-, -(CH_2_)_2_- and -CH_2_- vibrations. ^29^Si NMR spectrum and ^13^C NMR spectrum (Supplementary Materials Figure S1) show the typical features of the organosilica network with the efficient incorporation of both bis(triethoxysilyl)ethane and bis[triethoxysilyl(propyl)]tetrasulfide.

**Figure 2:**
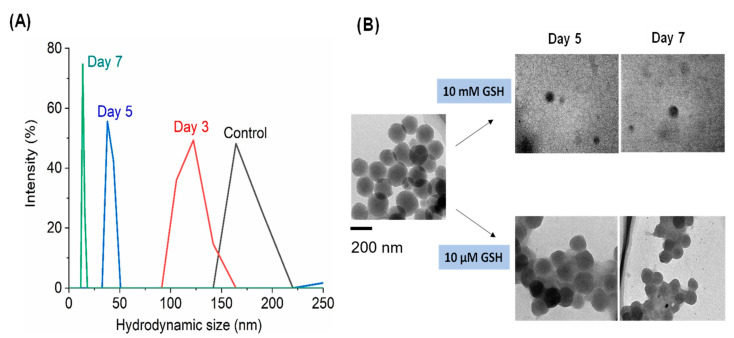
Degradation of BPMO nanoparticles in the presence of 10 mM glutathione (GSH) was investigated by examining nanoparticle size by dynamic light scattering analysis (DLS) (**A**) as well as by TEM analysis (**B**). Little degradation was observed after incubation with 10 μM GSH.

Figure 2A shows the degradation of BPMO in the presence of 10 mM GSH. As can be seen, the size of BPMO decreased to around 120 nm by day 3. By day 5, the size was less than 40 nm, and by day 7, the size was less than 20 nm. Figure 2B shows the TEM analyses. BPMO appears to be almost completely degraded by the incubation with 10 mM GSH for 5 days and for 7 days. In contrast, incubation with 10 μM GSH does not cause significant degradation.

### 2.2. Loading BPA onto BPMO Nanoparticles

**Figure 3:**
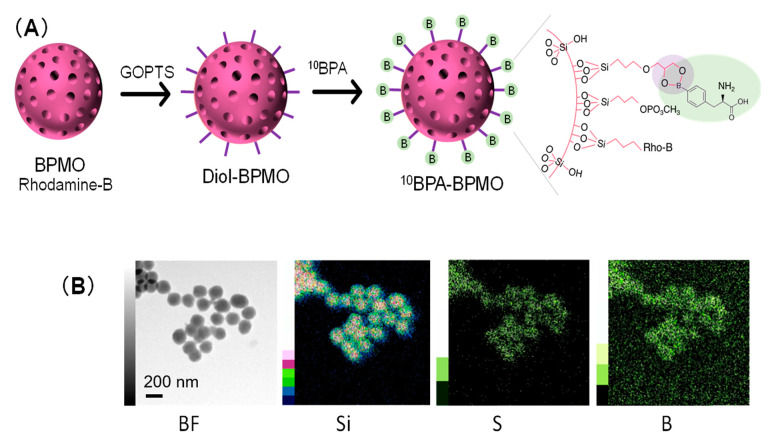
Loading of ^10^BPA to BPMO. (**A)** A strategy to load ^10^BPA (highlighted in green) to BPMO nanoparticles; (**B**) EDX-TEM analysis of ^10^BPA-loaded BPMO nanoparticles.

Because of the mesopore structure, BPMO has a large surface area [1]. To take advantage of this feature, we explored the ability of BPMO to load BPA by using a method to chelate boron by the use of diol groups on the surface. This method described in Figure 3A is based on a method developed to prepare a membrane that can capture boron in the water supply [26]. Briefly, BPMOs were processed to carry out postsynthesis grafting with GOPTS (3-glycidyloxypropyl trimethoxysilane). After converting epoxy groups to diols, the nanoparticles were incubated with ^10^BPA. Phosphonate surface modification was also carried out to keep the surface charge negative. The Zeta potential of BPA-BPMO was −42.48 mV (Supplementary Figure S2). 

The amount of boron loaded onto the nanoparticle was first examined by TEM elemental mapping analysis (energy dispersive x-ray spectroscopy-TEM). As shown in Figure 3B, the presence of boron, in addition to Si and S, was observed. To further confirm boron’s presence, ^10^BPA-BPMO was dissolved in nitric acid (HNO_3_) and then examined by ICP-OES (Inductively Coupled Plasma Optical Emission Spectroscopy), which revealed that boron constitutes 2.5% of the nanoparticle weight. The SEM and TEM analyses showed that ^10^BPA-BPMO have a diameter similar to that of BPMO, and the preparation was homogeneous. 

### 2.3. BPA-BPMO Are Efficiently Taken up into Cancer Cells and Are Localized to the Perinuclear Region

**Figure 4:**
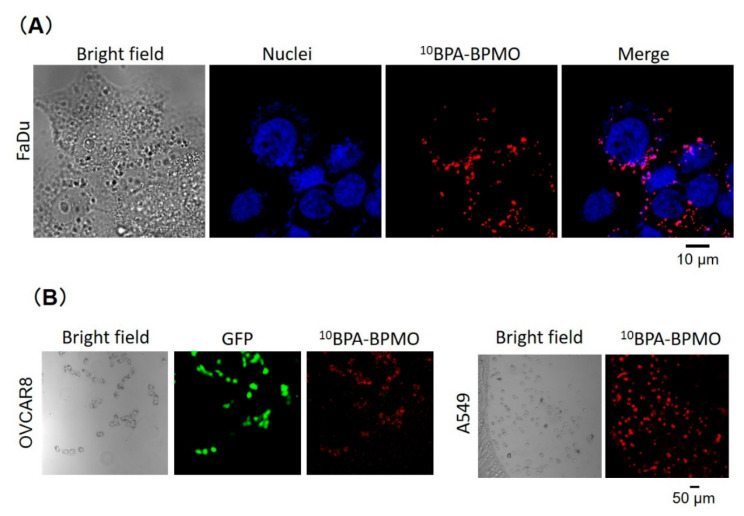
Cellular uptake of BPA-BPMO. (**A**) Confocal microscopy images of FaDu cells after incubation with BPA-BPMO. Images of bright field, nuclear staining with Hoechst dye, and red fluorescence of BPA-BPMO nanoparticles are shown. The rightmost picture shows overlay images of nuclei and nanoparticles; (**B**) Uptake of BPA-BPMO into OVCAR8 and A549 cells.

To examine how ^10^BPA-BPMO behaves in biological settings, we first used cancer cells and examined cellular uptake. We have observed the uptake of ^10^BPA-BPMO into a variety of human cancer cells, including ovarian cancer OVCAR8, lung cancer A549, and head and neck cancer FaDu cells. Figure 4A shows uptake into FaDu. As can be seen, red fluorescence of ^10^BPA-BPMO can be observed inside the cells. Nuclear staining with Hoechst (blue) was carried out, which revealed that the localization of ^10^BPA-BPMO is perinuclear. We have also examined the uptake into other types of cells. Figure 4B shows the uptake into the OVCAR8 and A549 cells. In the case of the OVCAR8 cells, they exhibit green fluorescence as these cells express green fluorescence protein (GFP). The red fluorescence of the nanoparticles overlaps with the green fluorescence. In the case of A549 cells, red fluorescence is detected inside the cells. These results show that BPA-BPMO is taken up into a variety of cancer cells. The cellular uptake of ^10^BPA-BPMO was also examined by flow cytometry (Supplementary Materials, Figure S3) which demonstrated the appearance of fluorescent cell population. In addition, confocal microscopy analysis showed that most cells have taken up the nanoparticles (Supplementary Materials, Figure S4). These results are consistent with our previous observation that mesoporous silica-based nanoparticles are efficiently taken up by endocytosis mechanisms [27]. In addition, there is involvement of LAT1 (L-type / large neutral amino acid transporter 1), an amino acid transporter, in the uptake, as BPA binds LAT1 [28]. This is discussed later (Discussion Section). 

### 2.4. BPA-BPMO Exhibit Tumor Accumulation in the Chicken Egg Tumor Model

**Figure 5:**
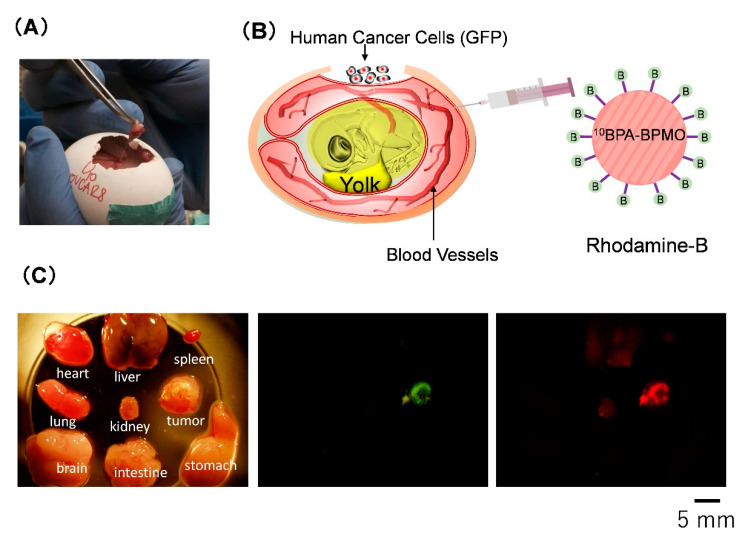
Characterization of nanoparticles by using the chorioallantoic membrane (CAM) assay. (**A**) Tumor formation on the CAM by transplanting cancer cells; (**B**) A CAM-based assay to examine tumor accumulation of nanoparticles. The tumor is formed using green fluorescence protein (GFP) expressing cells; (**C**) Detection of ^10^BPA-BPMO in tumor and organs.

We then used the CAM model to gain further insight into the action of BPA-BPMO in biological settings. The CAM model provides a versatile and convenient animal model to characterize nanomaterials [26]. This model uses fertilized eggs that have been incubated for ten days, at which time the embryo is surrounded by a nutrient-rich membrane called the chorioallantoic membrane (CAM). Human ovarian cancer cells OVCAR8 expressing GFP were transplanted onto the CAM. A tumor was formed in five days, as shown in Figure 5A. We have previously reported that the CAM tumor resembles a tumor from ovarian cancer patients [7]. 

We devised a simple assay shown in Figure 5B to evaluate the tumor accumulation of the nanoparticles. In this assay, we use OVCAR8 cells expressing GFP. Thus, the CAM tumor exhibits green fluorescence. Red fluorescent BPMO nanoparticles were intravenously injected. After two days, the tumor and various organs from the chick embryo were cut out, and the overlap of red fluorescence with green fluorescence was examined. A typical result is shown in Figure 5C. As can be seen, a significant amount of red fluorescence was detected in the tumor that was visualized by green fluorescence. Red fluorescence was also detected in the kidney and liver, but the extent was less than that seen in the tumor. Preferential tumor accumulation of ^10^BPA-BPMO was further confirmed by preparing thin sections of tumor and organs and examining them by confocal microscopy (Supplementary Materials, Figure S5). The tumor accumulation of BPA-BPMO appears to depend on many factors that contribute to prolonged circulation and enhanced permeability retention (EPR), as reviewed in the Discussion Section.

### 2.5. Irradiation of BPA-BPMO Accumulated CAM Tumor with Thermal Neutron Induces Tumor Growth Inhibition

**Figure 6:**
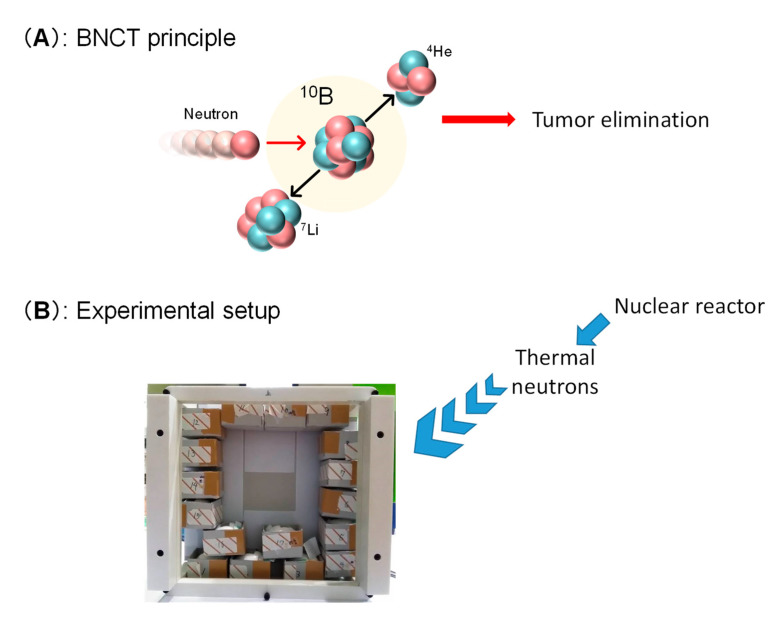
Boron neutron capture therapy (BNCT) principle and experimental setup. (**A**) BNCT principle involves irradiation of boron-10 with thermal neutron; (**B**) Egg holders were placed in a cardboard box and irradiated with thermal neutron from a nuclear reactor. The neutron beam was irradiated from the back of the cardboard box.

**Figure 7:**
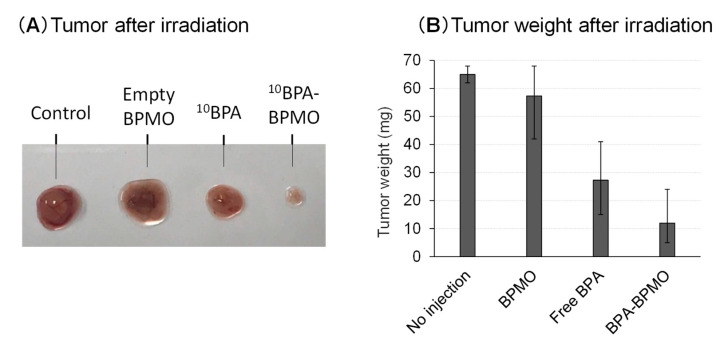
Tumor size after BNCT experiments. (**A**) Pictures of CAM tumors three days after neutron irradiation are shown; **(B**) CAM tumors were cut out after irradiation and their weights were examined.

Encouraged by the excellent tumor accumulation of BPA-BPMO in the CAM model, we then tested whether the CAM tumor’s thermal neutron exposure results in tumor growth inhibition. This experiment requires a neutron beam, and this was made possible by carrying out experiments at the Kyoto University Research Reactor, Heavy Water Neutron Irradiation Facility [29]. At this facility, “energetic” neutrons generated at the nuclear reactor core have their energy reduced to generate “thermal” neutrons. We placed tumor-containing chicken eggs that had been injected intravenously with ^10^BPA-BPMO at the center of the emerging neutron beam. A cardboard box that can hold 17 eggs was prepared (Figure 6B). The eggs were placed in such a way that each egg would receive a similar amount of neutrons. The thermal neutron exposure on each egg was approximately 3 × 10^12^/cm^2^. After being irradiated for one hour, the eggs were moved to an incubator and incubated at 37 °C. After three days, the size and weight of the tumors were examined. The results presented in Figure 7A and Supplementary Materials Figure S6 showed dramatic inhibition of the tumor growth when ^10^BPA-BPMO were injected. On the other hand, when empty BPMO with no ^10^BPA loading were injected, the tumor size was similar to that of the control (no injection). Free ^10^BPA inhibited tumor growth but was not as effective as that of ^10^BPA-BPMO. 

The results of the tumor weight measurement are shown in Figure 7B. The weight of the tumor after the neutron exposure with the ^10^BPA-BPMO administered sample was around 12 mg, while the tumor weight of control samples (no injection or injection of empty BPMO) was around 60 mg. Thus, 80% inhibition was observed with ^10^BPA-loaded BPMO. Tumor weight after the irradiation of the samples with free ^10^BPA was around 30 mg. We conclude that BPMO loaded with ^10^BPA significantly inhibits tumor growth after neutron irradiation. During the experiments, all eggs survived. 

## 3. Discussion

In this work, we have described the synthesis and characterization of BPA-loaded BPMO nanoparticles by employing surface modification of BPMO with diols that can be used to capture BPA. This synthesis was adapted from a method to prepare MSN membranes used to capture boron from the water supply. We further surface modified the BPMOs with phosphonate to increase their dispersibility. The BPMOs we used in this study had a diameter of 170 nm. However, various sizes of nanoparticles, ranging from 80 nm to 300 nm, can be synthesized by changing synthesis conditions. 

We have shown that BPA-BPMOs are taken up into cancer cells and are localized to perinuclear regions. This perinuclear localization may be important in placing boron-10 adjacent to the nucleus where DNA resides and may result in enhancing the effect of α-particles to cleave DNA, creating double-strand breaks that are highly lethal to cells. The cellular uptake appears to involve both general endocytosis and LAT1-mediated endocytosis. We have previously shown that our nanoparticles are efficiently taken up into a variety of cells by general endocytosis [27]. BPA is taken up into cells by LAT1-mediated transport [28] and potentially by LAT1-mediated receptor endocytosis when it is part of a larger macromolecule such as PVA-BPA [17]. To evaluate the involvement of LAT1-mediated endocytosis in the cellular uptake of BPA-BPMO, we examined BPA-BPMO uptake into FaDu cells in the presence of BCH (2-amino-2-norbornanecarboxylic acid), an inhibitor of the LAT1 transporter. The results showed that the BPA-BPMO uptake was decreased by approximately 40% compared to that seen in the absence of the inhibitor (data not shown). Thus, both general endocytosis and receptor-medicated endocytosis appear to be involved in the uptake. Further experiments are needed to investigate this point. 

In the CAM model, BPA-BPMO nanoparticles exhibit significant tumor accumulation. The presence of the nanoparticles in kidney and liver was also detected, but the level of this was less than that seen with the tumor. This preferential tumor accumulation appears to be influenced by a number of factors, including surface charge, the size of the nanoparticles, and the amount of BPMO injected as well as the time of incubation. We have previously shown that the surface charge is one of the key determinants of tumor accumulation; negatively charged BPMO tends to exhibit tumor accumulation while positively charged BPMO distributes to various organs [8]. The long circulation time required to achieve tumor accumulation (two days) suggests that prolonged circulation in the blood vessels is important, consistent with the idea that nanoparticles accumulate in the tumor by taking advantage of tumor vessels (EPR effect [30]). 

The exposure of tumor-bearing chicken eggs injected with ^10^BPA-BPMO to thermal neutron resulted in an 80% inhibition of the tumor growth. The inhibition was more pronounced compared with free ^10^BPA, demonstrating the advantage of using the nanoformulated boron reagent. This may be due to a preferential BPA accumulation in the tumor. These results point to the potential advantages of our BPA-BPMO over the current drugs used in BNCT therapy. Future development of new reagents that could be used with onetime administration and that have increased tumor accumulation will be valuable for BNCT cancer therapy. Further preclinical studies are needed to investigate these points. 

## 4. Materials and Methods 

### 4.1. Synthesis of BPMO

Synthesis of BPMO was carried out according to the procedure previously reported [8] with minor modification. Rhodamine B isothiocyanate (RBITC, 2.5 mg, 4.6 × 10^−3^ mmol) was dissolved in ethanol (EtOH, 5 mL), and then 3-aminotriethoxysilane (APTES, 6 μL, 2.6 × 10^−2^ mmol) was added. After stirring this solution for 30 min, 1,2-bis(triethoxysilyl)ethane (300 μL 8 mmol) was added for further 5 min. A mixture of cetyltrimethylammonium bromide (CTAB, 250 mg, 0.7 mmol), distilled water (120 mL), and NaOH (8M Sodium hydroxide, 219 μL) was prepared in a flask, stirred vigorously and then heated to 80 °C. After the CTAB solution reached a stable temperature at 80 °C, the silane containing solution was added dropwise into the flask. Then, bis[3-(triethoxysilyl) propyl] tetrasulfide (100 µL, 0.2 mmol) was added immediately. After condensation at 80 °C for 2 h, the material was collected by centrifugation (30 min at 14k rpm) and washed twice with EtOH. CTAB was removed from the pores by refluxing overnight in an ethanolic solution of ammonium nitrate (0.3 g in 50 mL). The particles were then purified by washing with EtOH (3 times) followed by desiccation. The purified materials were stored at room temperature for further characterization. BPMO: ^29^Si NMR (119 MHz, δ, ppm): -58.5 (T^2^), -66.9 (T^3^). ^13^C NMR (151 MHz, CP-MAS, δ, ppm): 5.0, 11.4, 23.1, 41.8, 64.2. IR (ν, cm^−1^): 1020 (Si-O-Si), 2891 (CH_2,s_), 2927 (CH_2,as_.).

### 4.2. Synthesis of Diol Modified BPMO

Dried BPMO (255 mg) was immersed in toluene (12.5 mL), and the mixture was sonicated for 10 min. After that, 3-glycidyloxypropyl trimethoxysilane (GOPTS, 2.1 mL, 9.51 mmol) was added to this mixture and refluxed overnight. The solid product was collected by centrifugation and washed 3 times with ethanol. The dried product was obtained and added to deionized water (30 mL). This mixture was heated at 70 °C for 2 days to open epoxide and convert it to diols. Diol-BPMO was further modified to confer a negative charge on the surface by stirring with 3-(trihydroxysilyl) propyl methyl phosphonate (220 μL, 1.00 mmol) in water (42 mL) at 70 °C overnight. 

### 4.3. ^10^BPA Loading

^10^BPA loading to Diol-BPMO was carried out by adding the Diol-BPMO (10 mg) into 1mL ^10^BPA solution (0.14 M, 29.14 mg into 1 mL) and deionized water (4 mL, pH 11.4); pH was then adjusted to 8.8-9.0 by the addition of 1 M HCl, and the mixture was stirred at 4 °C for 24 h. Nanoparticles were collected by centrifugation and then washed once with deionized water and twice with EtOH. The dried product was obtained after evaporation. ICP-OES was used to quantitate the amount of ^10^BPA loaded onto BPMO. For this measurement, ^10^BPA-BPMO was treated with 3M nitric acid for more than 20 h. After confirming that all the materials were dissolved, the sample was processed for ICP-OES. Comparison with standard boron curve revealed 5.47 ppm of boron in a 10 mL solution containing 2 mg of nanoparticles. We estimate that boron constitutes 2.5% of the weight of nanoparticle.

### 4.4. Characterization Methods

SEM (JSM-75FCT, JEOL, Tokyo, Japan) and TEM (JEM-2200FS, JEOL, Tokyo, Japan) analyses were performed on a microscope, respectively. JEOL JEM-2200FS+JED2300T system operated at 200 kV was used for the scanning transmission electron microscopy-energy dispersive X-ray (STEM-EDX) analysis. Powder X-ray diffraction patterns were collected on a X-ray Bruker D8 Advance system. Bruker E400 FT-IR spectrometer using potassium bromide pellets was used to measure FT-IR spectra. Low-pressure N_2_ adsorption measurements were carried out at 77 K on a Quantachrome Autosorb iQ volumetric gas adsorption analyzer (Helium was used as an estimation of dead space). Throughout adsorption experiments, ultrahigh-purity-grade N_2_, and He (99.999% purity) was used. DLS was performed using a Zetasizer μV Malvern apparatus (ZMV2000, Malvern Panalytical, Malvern, UK). We recorded the ^13^C and ^29^Si CP-MAS NMR spectra on a 600 MHz solid state NMR spectrometer (JNM-ECZ600R, JEOL, Tokyo, Japan) at a 20 kHz spinning rate. All confocal laser microscopy images were collected on a Nikon A1R confocal laser microscope (A1R, Nikon, Tokyo, Japan). The amount of boron in the samples was quantified using an ICP-OES emission spectrometer (ICPS-8100, Shimadzu, Kyoto, Japan). Degradation of BPMO in vitro was carried out by incubating BPMO in Phosphate-buffered saline (PBS) containing 10 mM GSH. After various incubation times, an aliquot was examined by DLS. 

### 4.5. Cell Culture and Nanoparticle Uptake

FaDu cells and A549 cells were obtained from ATCC and KAC co., Ltd., respectively. OVCAR8-GFP cells were provided by Dr. Carlotta Glackin (City of Hope Cancer Center). Initially, cells (1 × 10^6^) were cultured in a dish; after culture expansion, 5000 cells were seeded in a 35 mm culture dish. Twenty-four hours later, we added 25 µg/mL of ^10^BPA-BPMO (20 µL) per culture dish. Then, after 24 h of incubation, cells were washed with PBS three times. Cells were then fixed in 200 µL of 4% paraformaldehyde overnight and washed with PBS three times. Hoechst stain (200 µL of a 1:1000 solution) was used to stain the nucleus for 30 min at room temperature. Before confocal observation, the cells were washed with PBS twice, and suspended in 200 µL of PBS. FaDu cells (3 × 10^5^) were used for flow cytometry analysis. After 24 h of incubation at 37, 100 µg/mL ^10^BPA-BPMO (Rhodamine 6G labeled) was added to the cells. Then next day, the cells were washed with PBS three times. Then Hoechst dye was used for nucleus staining followed by two washes with PBS. Then cells were collected using 2.5% Trypsin solution. Before flow cytometry analysis, cells were filtered by a 50 µm sterile filter. SH800S Cell Sorter (Sony Biotechnology, Tokyo, Japan) was used with 405 nm excitation lasers for Hoechst and 561 nm laser for Rhodamine.

### 4.6. Chicken Egg Tumor Model (CAM Model)

Fertilized chicken eggs were purchased and incubated for 10 days at 37.5 °C under 65% humidity. Eggs were occasionally rotated. Chicken eggs were checked for the presence of thick blood vessels under the light, and a window adjacent to the blood vessels was opened with a grinder. Human ovarian cancer cells OVCAR8 expressing GFP (OVCAR8-GFP) were grown on 100 mm culture dish in RPMI1640 medium supplemented with 10% FBS (Fetal Bovine Serum) and 1% penicillin/streptomycin and used for transplantation. To transplant OVCAR8-GFP cells, a Teflon ring was placed on the CAM membrane, and 2.0×10^6^ of OVCAR8-GFP cells were added inside the Teflon ring. The OVCAR8-GFP tumor formed rapidly 3-5 days after the transplantation as previously described [26]. All chicken egg experiments were approved by the Kyoto University Animal Research Committee and were performed in compliance with the committee guidelines. In vivo experiments do not require any special additional allowance as long as the embryos are sacrificed before hatching, as was done in this study.

### 4.7. Tumor Accumulation of ^10^BPA Loaded BPMO in the CAM Model

0.2 mg of ^10^BPA-BPMO (suspended in 100 μL of sterile water and sonicated) was administered intravenously into the chicken egg, and biodistribution of BPMO was examined by red fluorescence of Rhodamine-B labeled BPMO by using a fluorescent stereomicroscope. Tumor and organs were fixed with 4% paraformaldehyde overnight at 4 °C. 

After washing with ice-cold PBS, tumor and organs were treated with 99.8% methanol for 30 min at -80 °C and washed once again in ice-cold PBS. Tumor and organs were then incubated overnight in a 20% sucrose solution at 4 °C. Sections (30 μm) were prepared and then stained with Hoechst 33258 stain diluted 500-fold with PBS, for 30 min in the dark. The BPMO biodistribution on thin sections was observed by using a confocal laser microscope.

### 4.8. Neutron Exposure Experiments

Fertilized eggs with OVCAR8-GFP tumor were injected intravenously with ^10^BPA-BPMO. After tumor accumulation (36 h after injection), they were exposed to a neutron beam. Eggs were irradiated for 1 h with thermal neutron beam at Kyoto University Research Reactor (KUR1), Heavy Water Neutron Irradiation Facility [29] at an operating power of 1MW. After the irradiation, the eggs were returned to the incubator and incubated for three days in 65% humidity. Tumors were then cut out, photographed, and weighed. For the injection of free BPA, an equivalent amount of BPA (prepared by solubilizing in alkali solution with fructose and then neutralized) was injected intravenously, and the eggs were exposed to a neutron beam. The thermal neutron exposure on each egg was approximately 3 × 10^12^/cm^2^ and each egg received similar amounts of neutron exposure. As a control, empty BPMO with no BPA loading was used. 

## Data Availability

The data presented in this study will be openly available.

## Supplementary Materials

Supplementary materials can be found at https://www.mdpi.com/1422-0067/22/5/2251/s1.
