# Supplementary material for: Construction of Boronophenylalanine-Loaded Biodegradable Periodic Mesoporous Organosilica Nanoparticles for BNCT Cancer Therapy
Source: Int J Mol Sci. 2021 Feb 24;22(5):2251. doi: 10.3390/ijms22052251 (PMC7956258; doi:10.3390/ijms22052251)
Supplement: Supplementary file 1 [file ijms-22-02251-s001.pdf]

Supplementary Materials

Construction of Boronophenylalanine-loaded Biodegradable Periodic Mesoporous Organosilica Nanoparticles for BNCT Cancer Therapy

Fuyuhiko Tamanoi[1]*, Shanmugavel Chinnathambi[1], Mathilde Laird[1], Aoi Komatsu[1], Albane Birault[1], Takushi Takata[2], Tan Le-Hoang Doan[3], Ngoc Xuan Dat Mai[3], Arthur Raitano[4], Kendall Morrison[4], Minoru Suzuki[2] and Kotaro Matsumoto[1]

[1] Institute for Integrated Cell-Material Sciences, Institute for Advanced Study, Kyoto University, Kyoto 606-8501, Japan; tamanoi.fuyuhiko.2c@kyoto-u.ac.jp
[2] Institute for Integrated Radiation and Nuclear Science, Kyoto University, Kumatori, Japan; suzuki.minoru.3x@kyoto-u.ac.jp
[3] Center for Innovative Materials and Architectures (INOMAR), Vietnam National University Ho Chi Minh City, Vietnam
[4] TAE Lifesciences, Drug Development Division, Santa Monica, CA 90404, USA
* Correspondence: tamanoi.fuyuhiko.2c@kyoto-u.ac.jp; Tel.: +81-75-753-9856

1. ^{29}Si NMR spectrum and ^{13}C NMR spectrum

^{29}Si NMR spectrum of BPMO is shown in Figure S1A. As can be seen, typical features of organosilica network with signal at -58.5 (aliphatic T^2) and -66.9 (aliphatic T^3) respectively were detected. No Si-C cleavage could be observed as evidenced by the absence of Q-type signals around -100 ppm. In the ^{13}C NMR spectrum (Figure S1B), the

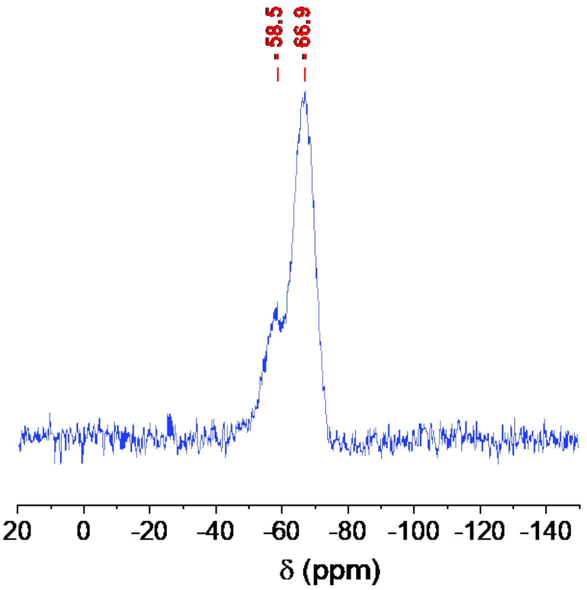

Figure S1A. ^{29}Si CP-MAS solid state NMR spectrum of BPMO

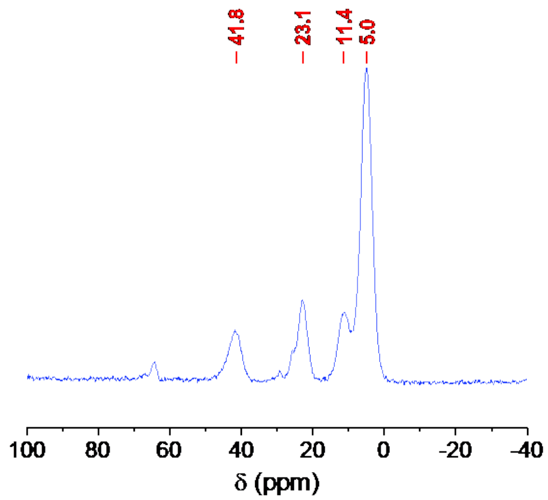

Figure S1B. ^{13}C CP-MAS solid state NMR spectrum of BPMO

signals arising from both bis-triethoxysilylethane at 5.0 ppm and bis(triethoxysilylpropyl)tetrasulfide at 11.4, 23.1, 41.8 ppm show the integration of both precursors on the silica network. Given the low quantity of Rhodamine B derivative used in this synthesis, no signal attributed to the later could be clearly observed.

2. **Surface charge of BPA-BPMO**

BPA-BPMO were dispersed in 1mL of deionized water and sonicated for 10 minutes before zeta potential measurement. The zeta potential of BPA-BPMO was measured by Zeta potential/particle size measurement system ELSZ-2000ZS (Otsuka Electronics Co., Ltd., Japan) at room temperature. The zeta potential of BPA-BPMO shows -42.48 mV (Figure S2).

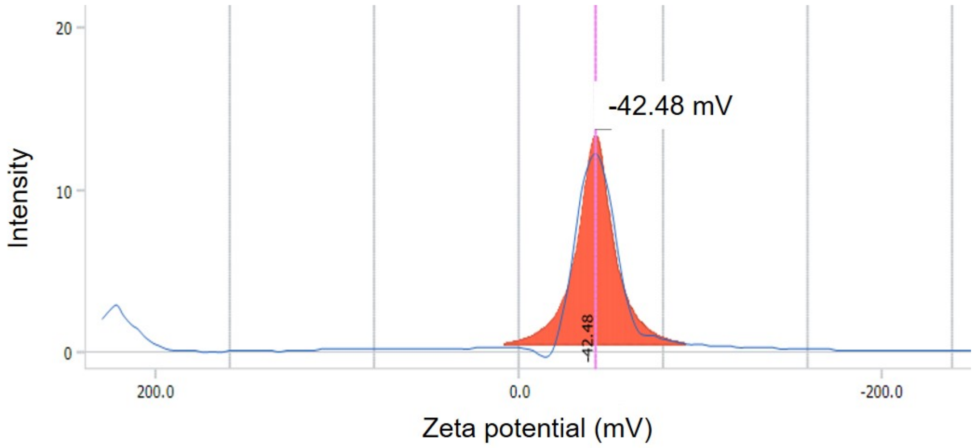

Figure S2. Surface charge of BPA-BPMO measured by zeta potential.

3. **Uptake of ^{10}BPA-BPMO into FaDu and OVCAR8 cells**

Uptake of ^{10}BPA-BPMO into FaDu cells was examined by flow cytometry (Figure S3). Cells were incubated with ^{10}BPA-BPMO (Rhodamine labeled) for 12 hours and then

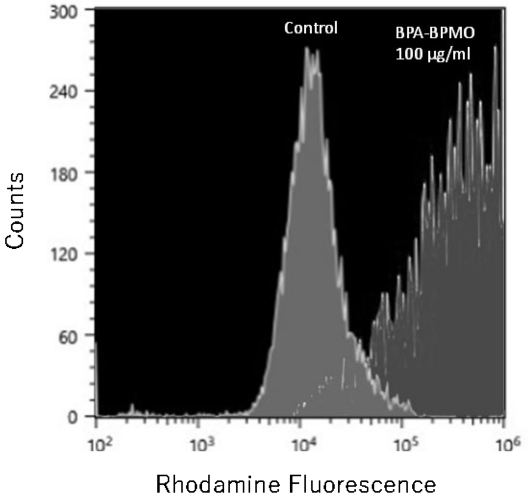

Figure S3. Flow cytometry analysis of FaDU cells incubated with ^{10}BPA-BPMO.

analyzed by flow cytometry. Shift of the fluorescence indicates uptake of nanoparticles. The ratio of FaDu cells which had taken up Rhodamine B-labeled ^{10}BPA-BPMO was calculated to be 63 %.

We also examined uptake of ^{10}BPA-BPMO into OVCAR8 cells by confocal microscopy (Figure S4). Cells were incubated with 0-50 μg/ml ^{10}BPA-BPMO and the presence of red fluorescence was examined by confocal microscopy. Cell nuclei were labeled with Hoechst dye. Percentage of cells that exhibit both red and blue fluorescence (number of cells examined: 50 for each group) was scored and plotted against the concentration of nanoparticles used. As can be seen, percent of cells that have taken up ^{10}BPA-BPMO approached 100% with high concentrations of the nanoparticle.

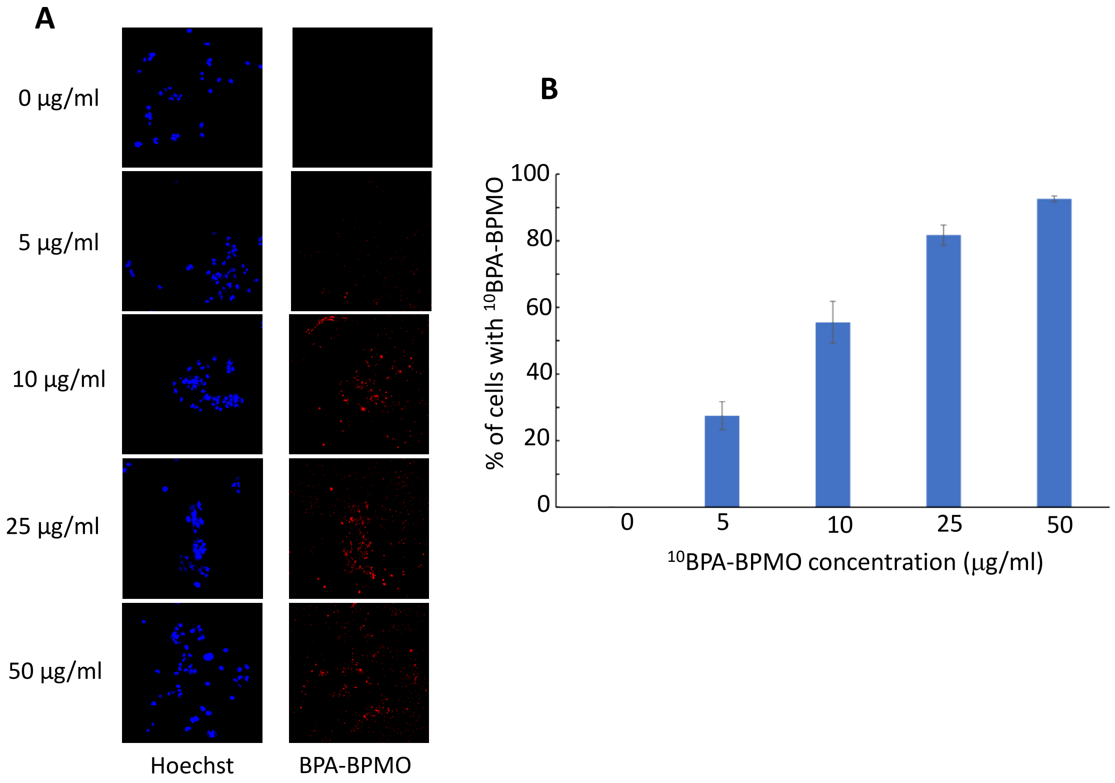

Figure S4. Uptake of ^{10}BPA-BPMO into OVCAR8 cells was examined by red fluorescence of Rhodamine-B labeled ^{10}BPA-BPMO. Cell nuclei were stained with Hoechst. (A) Confocal microscope pictures. (B) Percentage of cells that exhibit both red and blue fluorescences was examined and plotted against the concentration of the nanoparticle.

4. Accumulation of BPA-BPMO in the CAM tumor and organs

Tumor as well as heart, liver and kidney were collected two days after the injection and their fluorescence was detected by using a confocal microscope (Nikon First-Scan Confocal Microscope A1R) which was equipped with the 10×lens (CFI Plan Apo 10, Nikon). The wavelength of excitation (Exc) and fluorescence emission (Emis) was: GFP, Exc at 488 nm and Emis at 500-550 nm; Hoechst 33258, Exc at 405 nm and Emis at 425-475 nm; Rhodamine B, Exc at 561 nm and Emis at 570-620 *nm.* As can be seen in Figure S5, red fluorescence of BPA-BPMO was detected in tumor while detection in other organs (heart, liver and kidney) was low.

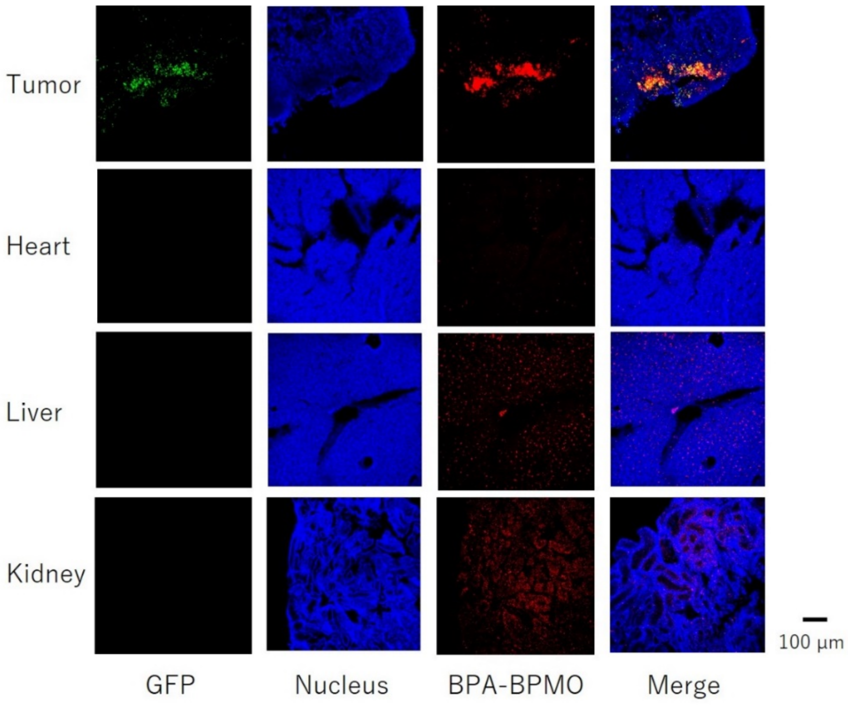

Figure S5. Confocal images of the accumulation of BPA-BPMO in the CAM tumor and organs.

5. Inhibition of tumor growth by neutron irradiation

CAM tumors injected with ^{10}BPA-BPMO, empty BPMO, ^{10}BPA were irradiated with thermal neutron and tumor weight was examined three days after the irradiation.

	Tumor weight (mg)
No injection	68, 62, 62
BPMO (empty)	62, 68, 42
Free BPA	18, 28, 41, 27, 34, 28
BPA-BPMO	13, 14, 14, 10, 6, 5

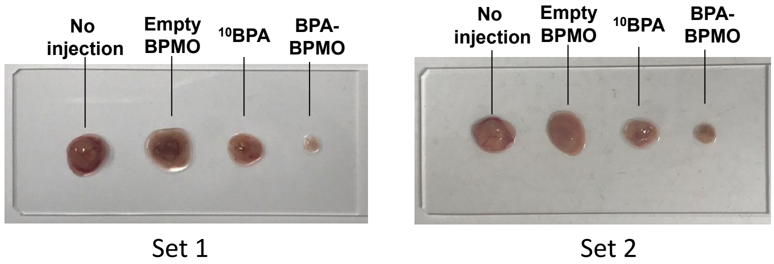

Figure S6. Tumor weight and tumor picture after neutron irradiation.

No injection was used as a control. Tumor in each chicken egg was cut out and the weight of each tumor was measured. This is indicated in Figure S6 top. Two sets of representative tumor pictures are shown in Figure S6 bottom.
